# Avoiding Animal Suffering and Preserving Human Lives: Mind Perception and Speciesism in Moral Judgments of Torture and Killing

**DOI:** 10.1177/01461672251367841

**Published:** 2025-10-15

**Authors:** Simon Myers, Jesse L. Preston, Adam Sanborn

**Affiliations:** 1 University of Warwick, Coventry, UK; 2 University of Kent, Canterbury, UK

**Keywords:** morality, harm, mind perception, speciesism, anthropocentrism

## Abstract

What is worse—torturing an animal or killing it? What about humans? In three studies (*n* = 472), torturing animals was perceived as worse than killing, but this was significantly reduced or reversed for humans. This was partially explained by mind-perception (agency or experience), and also by an aversion to the loss of human lives over and above this (speciesism). Study 1 provided evidence that the moral wrongness of torturing a hypothetical animal was worse than killing, but killing was worse for human targets. Study 2 partially replicated and extended these results across different species. Ratings were predicted by mind perception, and speciesist preference to avoid human death. Study 3 used pairs of species, separating torture and killing judgments, showing that while speciesism is important for explaining the greater weight people place on human lives, it played a smaller role in judgments about suffering after accounting for mind-perception.

Suppose that someone has captured an animal. They torture the animal for a while, purely for their own amusement, before finally letting it go. In contrast, consider someone who captures an animal but just wishes to kill the creature, again purely for their own amusement. They do so painlessly by shooting it through the head. Which of these acts is more morally wrong, the infliction of suffering without death or the painless taking of the animal’s life? Now, instead, suppose it is a human being who is captured as the target of harm. Is it worse to torture or to kill that person? If readers share the authors’ intuitions, the answer to this moral thought experiment changes when the victim is a human: torturing an animal seems more morally reprehensible than just killing it, but killing a person is much worse than just torturing. But why?

Philosophical and ethical theories differ on whether suffering or killing is worse, and in many instances, the answer depends on the victim. That is, some kinds of harm may be worse for some kinds of entities compared to others. In classical formulations of Utilitarianism, the concept of utility was centered on one’s ability to have valenced mental states like happiness and pain (Bentham, 1789; Mill, 1887; Sidgwick, 1981). Preference Utilitarianism, on the other hand, emphasizes the capacity for having preferences at different levels of sophistication (e.g., the preference to continue living), and immoral acts are those that frustrate those preferences (Hare, 1981; Singer, 1995, 2011). Kantians also require the entity to be a “rational being” where the agent’s intelligence and ability to think is the focus (Kant & Schneewind, 2002). The fact that humans have a larger array of more complex preferences compared to animals is considered integral to why we believe human life should be of particular importance (Singer, 2011). Much philosophical work has been spent trying to tease apart these different considerations, especially the contrasting moral weight of suffering and death for non-human animals, for a review see Višak and Garner (2016), with distinct moral theories weighing on each. However, less is known about the psychology that might underpin these judgments.

## Mind Perception and Moral Concerns for Harm

While philosophers provide normative reasons why the faculties of different moral targets should matter, psychologists have given us tools for assessing how we perceive and attribute those faculties to different entities. Intentional harms such as causing pain or death have been the focus of much work in moral psychology (Graham et al., 2009; Gray et al., 2014; Haidt & Joseph, 2008). A simple conception of intentional harm is that there is a single-dimensional scale of harm, with some harms universally worse than others (Schein & Gray, 2018). But in comparing extreme harms of torture without death versus death without pain, moral judgments are less clear.

As in philosophy, psychologists suggest that moral judgments of harm are informed by the perceived capacity and quality of mind. Gray et al. (2007, 2012) find that the kinds of minds people automatically attribute to different entities vary across the two dimensions of agency and experience. Agency relates to the ability to reason and act on the world (e.g., memory, planning, self-control) whereas experience relates to the ability to feel (e.g., pleasure, emotion), including the ability to feel pain. Importantly, here, various humans and other species vary greatly in their perceived capacity for agency and experience (Gray et al., 2007). Non-human animals are perceived as considerably less agentic than humans, that is, lacking higher intelligence and the capacity for complex thought. (Morewedge et al., 2007). But non-human animals are still credited with some capacity for basic emotion, though some types of emotion are seen as “uniquely human” to the extent that they are tinged with more complex or agentic aspects of feeling, for example, nostalgia (Demoulin et al., 2004).

Moreover, these dimensions of mind can be used to explain numerous aspects of moral psychology, including moral responsibility in artificial intelligence (Bigman et al., 2019), and religious conceptions of suffering (Gray & Wegner, 2010b). Targets of harm (or “moral patients”) that are high in capacities for experience are judged to feel more pain, but moral patients higher in agency are seen as better capable of enduring pain (Gray & Wegner, 2011). Judging torture has effects on both empathy and ascribing guilty where there is greater empathy for the victim, and those victims are seen as less guilty because one is more psychologically distant. Hypothetical animals seem more distant than a human and are given less empathy (Gray & Wegner, 2010a).

The emphasis of different dimensions of mind in humans versus animals holds direct implications for the relative moral judgments of torture or killing. Torture and killing are both extreme harms, but the dimensions of mind perception may differ in importance for judging this harm. Torture is an extreme harm because it is painful, and so can be considered a harm to the extent that the target has a capacity for experience. Without the capacity for pain, torture would not be torture. Death is also an extreme harm, robbing the target of their life and with it the potential to do anything (i.e., to use their agency to act on the world). In other words, the cessation of life is the cessation of agency. The link between experience and the moral wrongness of torture is likely to be clearer than the link between death and agency. For the latter, a target with high agency will have more ability to think and plan for the future, a future that would be extinguished if they were killed. That is, the agent is likely to have more complex forward-looking desires, preferences, and goals than their immediate hedonic concerns, all of which would go unfulfilled, making the prospect of their death potentially more concerning for those who care about thwarting the preferences of other beings as this would include a far greater set of preferences. Also, if the agent had a specific preference against death, this may require understanding fairly high-level concepts (such as the concept of death itself)—all of which require the more complex thinking that is associated with perceptions of high agency.

This fundamental difference suggests that the relative wrongness of these harms should follow from the recipients’ predominant dimension of perceived mind. Torture should seem especially bad for targets that are ascribed a strong capacity for feeling, and death should seem especially bad for targets ascribed with the strong capacity for agency.

## Speciesism and the Value of Human Life

Additionally, there may be some fundamental reasons why the loss of human life or human suffering would be treated differently. The preservation of human life is often taken as a sacred (Tetlock, 2003), and a guiding principle in conversations about capital punishment, healthcare, abortion, and war (Atran & Axelrod, 2008; Ginges & Atran, 2013). Devaluation of the sacredness of human life, by framing it in statistical or monetary terms, by governments or businesses, runs the risk of appearing callous (Rice & Cooper, 1967). The value of human life is so much taken for granted that it is seen as a goal in the development of any natural phenomena that benefit human life (e.g., trees produce oxygen so that humans can breathe), and this bias is resistant to correction by making similar advantages to other species salient (Preston & Shin, 2021). The idea that humans are in some sense exceptional and that human welfare must always categorically take precedence over the suffering of animals is a form of *anthropocentric speciesism*, that is, moral worth determined only on the basis of species membership, (Caviola et al., 2019, 2020; Fjellstrom, 2002; Horta, 2010). Anthropomorphism—attributing human characteristics to nonhumans—has positive effects on their moral treatment (Butterfield et al., 2012; Waytz et al., 2010). But though we may recognize in humans some unique features salient to moral concern, such as abstract thinking, social and familial ties, emotion and language, (Grau, 2016; Kagan, 2016), there is evidence for many of these features in the animal kingdom and they differ a great deal between the species (Gruen, 2017). Instead, people may hold an anthropocentric prejudice, likened to racial prejudice or sexism, purely on the basis of species membership and irrespective of the abilities they may (or may not) attribute to non-human animals (Caviola et al., 2019; Fjellstrom, 2002). In fact, speciesism better predicts moral worth judgments over and above the role of mind perception (Caviola et al., 2019). This anthropocentric speciesism can be seen in the extra deontological constraints placed on human lives compared to animal lives in sacrificial dilemmas (Caviola et al., 2020). For example, in trolley dilemmas, people are averse to sacrificing a human in order to save five other people (Greene, 2009; Kahane et al., 2018), but this constraint becomes more relaxed when sacrificial dilemmas instead involve animals, both hypothetical dilemmas (Caviola et al., 2020), and in laboratory experiments (Bostyn et al., 2018). Despite this, it appears that judgments regarding moral character are made more strongly for harming animals compared with harming people (Tannenbaum et al., 2011). Although many arguments have been made with regards to how we consider the value of animal lives, and their welfare (Višak & Garner, 2016), there is as yet less empirical work in the same vein.

## Present Research

While previous research makes predictions for people’s preferences for extreme harms, we are unaware of any previous empirical investigations that compare extreme harms of suffering versus killing across different human and animal targets. In three studies (*n* = 472), we test how people differentiate between moral targets in terms of suffering and killing. We predict that, overall, there will be an asymmetry in moral judgments of torture versus killing depending on whether the victim is human or a non-human animal. Furthermore, we explore the roles of mind perception (agency and experience) and speciesism as mechanisms to explain differences in moral intuitions of extreme harms. Specifically, our preregistered predictions, synthesized from the above literature, were as follows:

H1: All else being equal, killing will be judged as morally worse than torture for human targets.
H2: All else being equal, torture will be judged as morally worse than killing for non-human targets.
H3: Relative moral judgments of killing vs. torture may be partially explained by perceived differences in the agency vs. experience of the target.
H4: All else being equal, moral judgments of extreme harms will be stronger for human vs. non-human targets.

## Experiment 1

Experiment 1 served as an initial test of the intuition that judgments of the relative moral wrongness of torture versus killing differ for human versus animal targets. Participants viewed either an unspecified hypothetical animal or a hypothetical human as the target of a sadist who either wishes to torture or kill them. We predicted that torture would be seen as morally worse than killing when the victim is an animal (non-human), but that killing would be seen as worse than torture for human victims. This study was pre-registered: https://osf.io/t7zpn/?view_only=31beb985c8384f4fb6f74e344cba0077.

We report all manipulations, measures, and exclusions in this and the following studies.

### Participants

We recruited 149 participants from the United States and Canada (aged between 19 and 73, *M*_age_ = 35.45, 91 male and 56 female) from Amazon Mechanical Turk in exchange for payment of $2.40 USD. Participants were recruited based on the criteria that they must have participated in least 50 previously accepted studies with a prior approval rating above 90%. Sample size chosen based on previous research on mind perception. Post hoc sensitivity power analyses conducted with our final sample using G*power (Faul et al., 2007), with α = .05 and 1 − β = .80, yielded a minimum detectable effect size of *d* = 0.20 (for a one-sided *t*-tests against indifference—see one-sample *t*-tests below).

### Procedure

Questions for this study were attached to the end of an unrelated study (see details for that study, see its pre-registration here: https://osf.io/m5jrn/?view_only=1b6a841650f84e27a6cf8d3b12abdb0b).

In a two-group between-subjects design (human/ animal), participants read a description about two different individuals who either kill or torture for fun. The vignettes read as follows: “*Imagine there is a person, who for fun targets random [animals/people] and kills them instantly and painlessly by shooting them in the head. Now imagine there is a person, who for fun targets random [animals/people], captures them and for a short period and tortures them to inflict pain before letting them go. In general, out of the act of killing an [animal/human being] or torturing an [animal/human being], which do you feel is morally worse?”*

Participants judge whether the act of torture or the act of killing is worse on a 7-point scale ranging from (1) = *“torture is much worse*” to (7) *=* *“killing is much worse.*”

### Results

Analyses were conducted in Jamvoi V2.3 (Şahin & Aybek 2019), Bayesian priors were set to defaults unless indicated otherwise. The index BF_10_ refers to how strongly the data favors the alternative hypothesis compared to the null hypothesis where *BF*_10_ > 3 is interpreted as the data substantively favoring the alternative hypothesis, and a *BF*_10_ < ⅓ is interpreted as the data substantively favoring the null hypothesis. Values between ⅓ and 3 indicate that the test should be considered insensitive (Kass & Raftery, 1995).

**Figure 1.**
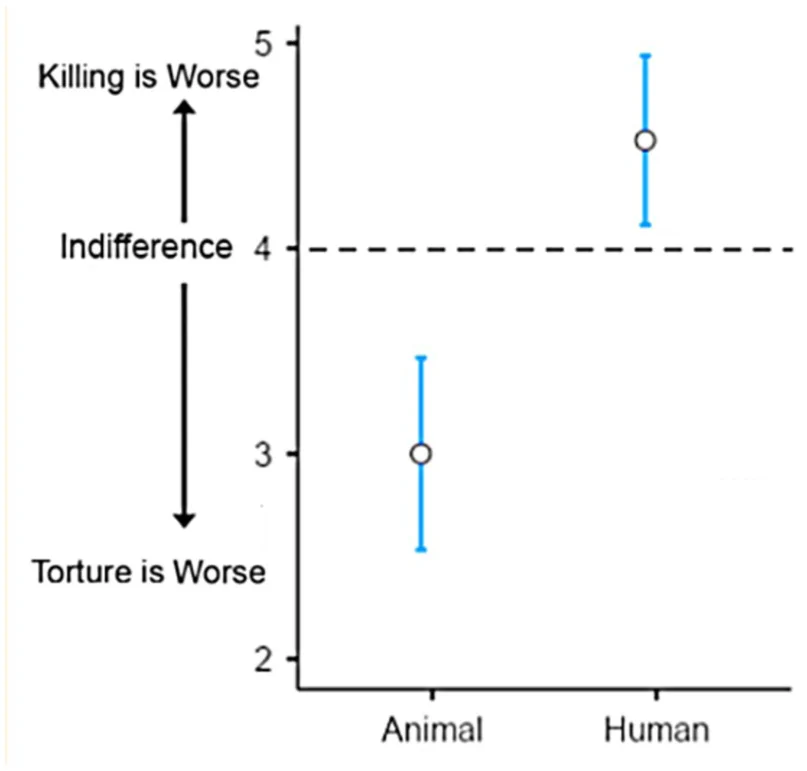
Moral wrongness of torture versus murder, by species-type (animals/humans), Study 1. *Note.* Points indicate the mean, and bars indicate 95% CIs.

Relative judgments of torture versus killing were made on a single 7-point scale, where the midpoint (4) reflects participants’ indifference or indecision between the two extreme harms. We first tested whether judgments from each group (human or animal targets) differed from indifference using two one-sample *t*-tests. For both animals and humans, killing and torture judgments were significantly different from indifference, but on opposite sides of the scale. As predicted, torture was judged to be worse than killing for animal victims, (*M* *=* 3.00, *SD* = 2.07), *t*(74) = −4.19, *p* < .001, *BF*_10_ = 216.47. But for humans, killing was judged as worse than torture (*M* = 4.53, *SD* = 1.81), *t* (73) = 2.51, *p* = .007, *BF*_10_ = 4.7, see Figure 1.

Moreover, we were also interested in whether an asymmetry in judgements over extreme harms exists between animals and humans. An independent samples, Welch’s *t*-test, revealed that judgments for animals and humans significantly differed from each other *t*(144.92) = 4.8, *p* < .001, *Cohen’s d* = 0.79, *BF*_10_ = 4,039.

### Discussion

Experiment 1 provided initial evidence for an asymmetry in judgments of extreme harms for humans versus animals. For animals, torturing is judged to be worse than killing, but for humans, the reverse pattern is observed. The experiments that follow test hypotheses that may explain this difference.

## Experiment 2

The goal of Experiment 2 was to replicate the general effect observed in Experiment 1, that is, a difference in relative wrongness of extreme harms (torture/kill) depending on the target of harm (human/animal). Experiment 2 extends the procedure used in Experiment 1, by asking for judgments across a selection of specific human and animal targets. Again, we predicted an asymmetry in moral judgments of torture versus murder that animals and humans will be treated differently in terms of comparing torture and murder, such that torture would be seen as worse for animals and killing would be seen as worse for humans. We also investigate differences in mind perception, and the capacities for experience (feelings, sensations) and agency (intelligence, self-control) as potential mechanisms for these asymmetric judgments in judgments for torture and killing. The study was pre-registered: https://osf.io/a65c7/?view_only=19cad232626042af9bcc1113d2815d1d

### Participants

We recruited 161 participants (aged between 18 and 28, *M*_age_ = 18.93, 16 male, 143 female) from the psychology undergraduate research experience pool at the University of Warwick. Participants received partial course credit for taking part in the experiment. Post hoc sensitivity with α = .05 and 1 − β = .80, yielded a minimum detectable effect size of *d* = 0.20 (for a one-sided *t*-tests against indifference—see one-sample *t*-tests below). In addition, we performed a simulation-based sensitivity analysis for the mixed model predicting judgments with Target-Type, agency, and experience as predictors and random intercepts for each participant. For 80% power, the determined minimum effective sample size was 60 participants (1,320 observations) for detecting the effect of agent type; 60 participants (1,320 observations) for detecting the observed effect of experience; and 150 participants (3,300 observations) for detecting the observed effect of agency.

### Procedure

To test whether differences in judgment exist only between humans and animals or whether differences exist between multiple different targets, we used a within-subjects design where participants made judgments across 22 targets (11 humans and 11 animals). The animals were: *octopus, crab, family dog, cow, sheep, chicken, orangutan, spider, ant, pigeon* and *police dog*. The humans were: *doctor, CEO, teacher, child, baby, athlete, warehouse worker, person with a severe mental disability, you—the participant, accountant*, and *bus driver*.

Each target was also rated on perceived mind. One item inquired about perceived agency: “*How much each of these targets is capable of thinking. That is, how much is each able to plan ahead, reason about ideas, think, and have general intelligence.”* One item asked about perceived experience: “*How much each of these targets is capable of feeling. That is, how much can they have emotions and feelings, and experience sensations of pain and pleasure.”* Both mind perception items were rated on five-point scales (endpoints: 1 = *not at all*, 5 = *extremely*). Finally, participants were asked which act was more morally wrong, on a six-point scale between torture and killing (endpoints: 0 = *torture is much worse*, 5 = *killing is much worse*). For exploratory purposes, we also coded animals based on whether or not they are farm animals and conducted the same analyses. The results remained qualitatively the same (see Supplemental Material).

### Results

**Figure 2.**
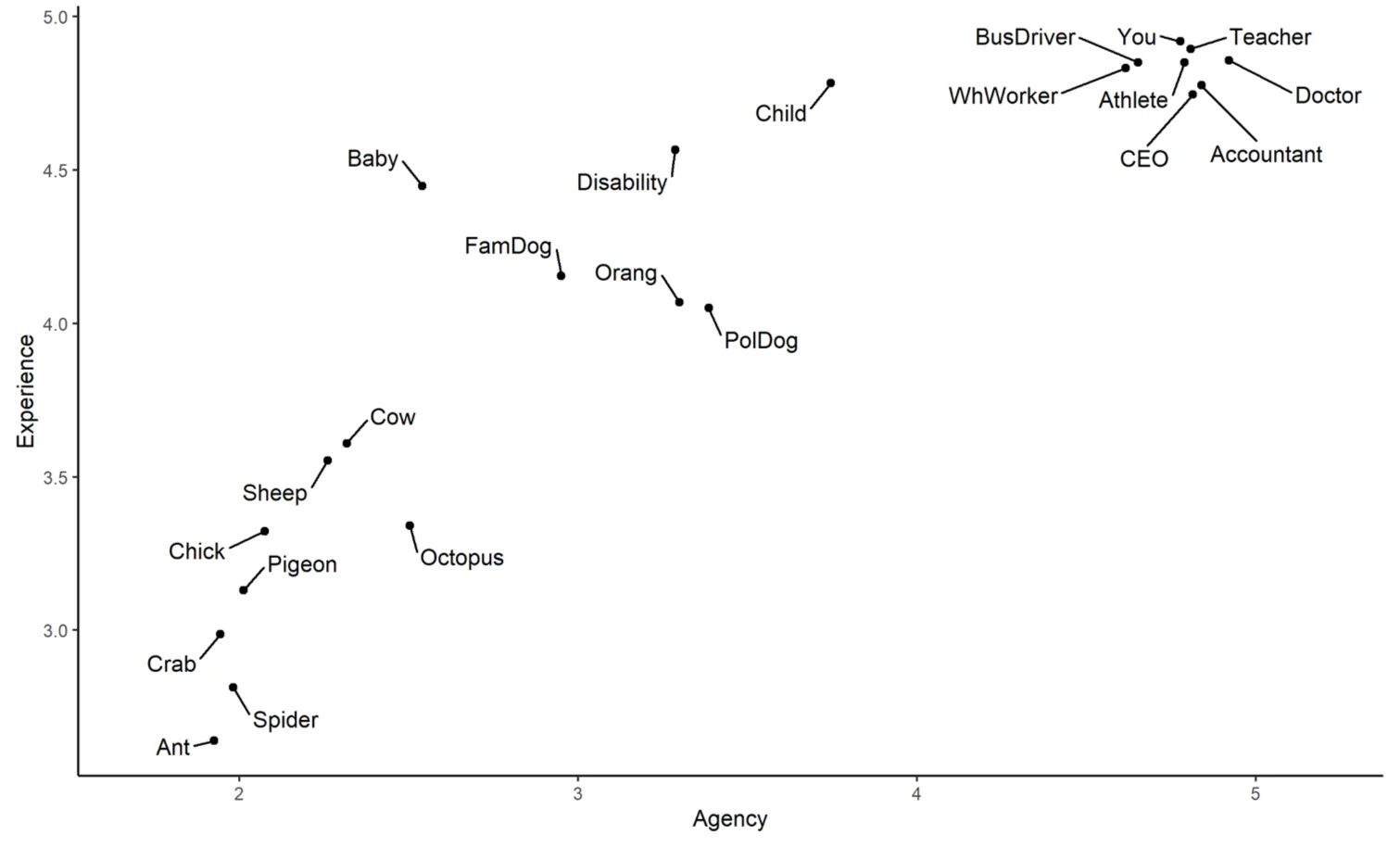
Mind perception (agency/experience) for each target, Study 2. *Note.* WhWorker = Warehouse Worker, FamDog = Family Dog, PolDog = Police Dog.

**Figure 3.**
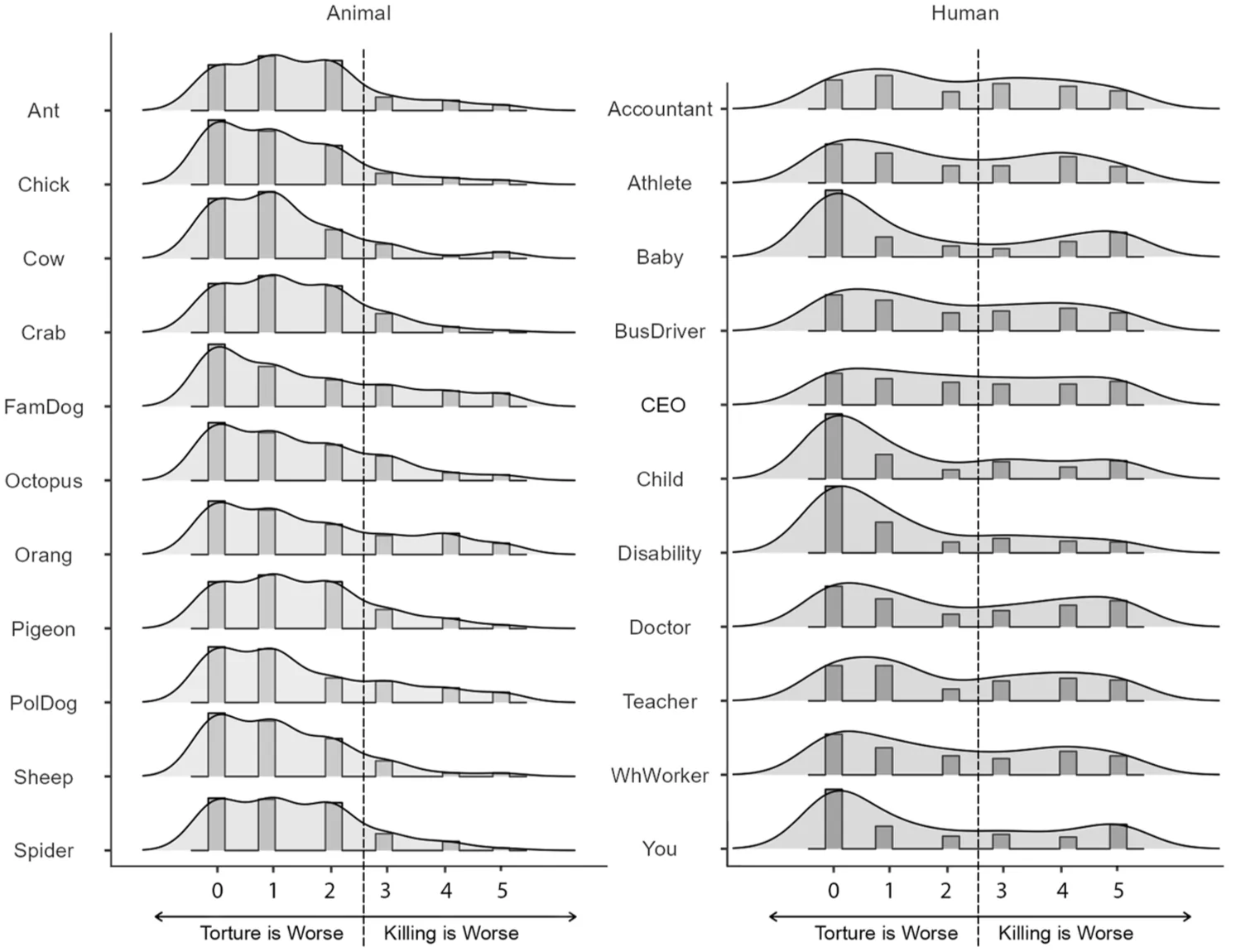
Preferences for torture versus murder by target. *Note*. WhWorker = Warehouse Worker, FamDog = Family Dog, PolDog = Police Dog.

To visualize how the targets differed in terms of mind perception, Agency and Experience were plotted for each target (see Figure 2). Interestingly, while there was a great degree of variability between animal targets, humans are mostly clustered as having both high agency and high experience. Notable exceptions were a child and a person with a severe mental disability, both were relatively lower on perceived agency. Although these measures of mind perception are highly correlated, *r* = .63, they are kept separate for the following analyses so that we can assess the hypothesis that each dimension differentially predicts judgments of torture versus killing. That is, keeping them apart (rather than using a composite measure) enables us to test whether it is the case that higher perceived agency predicts judgments that killing is worse than torture and higher perceived experience predicts judgments that torture is worse than killing. Figure 3 shows the distributions for Torture versus Killing.

To assess judgments comparing torture to killing for animals and for humans, two random effects models were calculated. These models generalized across target agents (random intercepts for target) and random intercepts by participant were also specified. Ratings were recoded so that zero represented indifference between torture and killing, allowing for tests of the intercept. Positive values meant killing is worse, and negative values meant torture was worse. For the animal group, the intercept was significantly lower than indifference, *b* = −1.10, *SE* = 0.09, *p* < .001; and for the human group, the intercept was also significantly lower than indifference, *b* = −0.54, *SE* = 0.15, *p* < .001. This indicates that for both groups torture was seen as worse, although this was true to a greater extent for animals as ratings were significantly lower *F*(1,20) = 28.9, *b* = 0.57, *SE* = 0.12, *p* < .001. We then decomposed this into separate one-sample *t*-tests to look at each target individually.

The one-sample *t*-tests showed judgments for all animal targets significantly differ from indifference falling on the “torture is worse side,” all *p*s < .001 (holm corrected). Judgments for human targets were more mixed. Against our prediction, 7 out of the 11 of the human targets (athlete, baby, bus driver, child, person with disability, warehouse worker, and “you”) also significantly differed from indifference but falling on the “torture is worse side” all *p*s < .001 (except warehouse worker *p* = .022). However, unlike the animal targets, distributions were more spread with torture being more clearly worse for human targets perceived to have the least agency (*child, baby, person with severe mental disability*), see Figure 3. Additionally, four of the human targets were not significantly different from indifference (accountant *p* = .138; CEO *p* = .151; doctor *p* = .138; teacher *p* = .06), meaning that for these targets, there was not a significant preference for one over the other. Equivalent Bayesian default one-sample *t*-tests were performed to assess the strength of evidence for each of these. The Bayes Factors were as follows, Doctor = 1.61; Accountant = 1.60; Teacher = 1.66 only anecdotally favoring the null (the test was insensitive); CEO = 4.11, substantively favoring the null. Importantly, the distributions indicate that participants judging harms to humans were more likely to say that killing is morally worse than torture, compared to those making judgments about animals, see Figure 3. Indeed, twice as many people rated killing as worse for humans than for animals.

Frequentist mixed models were calculated using the GAMLj module for Jamovi (Gallucci, 2019). Bayesian models (quantifying the levels of evidence for and against each predictor/model) were fitted using the Bayes Factor package with default settings (Morey et al., 2015). All mixed models initially specified all random slopes for all predictors, and then random slopes were removed one by one to find the maximal converging model, which is recommended as best practice for linear mixed-effects models (Barr et al., 2013). The maximal converging model was: Rating ~ Target Type + Agency + Experience + (1 + Target Type + Agency + Experience | Participant ID), meaning no random slopes needed to be removed for convergence^1^.

All Bayesian mixed models had random slopes specified for all predictors. The bayesTestR package was used for calculating Bayes Factors of inclusion (comparing all possible models with the term against all equivalent models with that term removed; Makowski et al., 2019).

The results indicate that Target-Type, Agency, and Experience were significant predictors of torture vs killing judgments. Target-Type, *F*(1, 164) = 12.6, *b* = 0.32, *SE* = 0.09, *p* < .001, *BF*_10_ = 35.53; Agency, *F*(1, 156) = 47.3, *b* = 0.21, *SE* = 0.03, *p* < .001, *BF*_10_ = 5.22 × 10^11^; Experience, *F*(1, 96) = 10.8, *b* = −0.12, *SE* = 0.04, *p* = .001, *BF*_10_ = 20.75. As predicted, the higher the perceived agency, the worse killing is compared to torture. In contrast, the higher perceived experience, the worse torture is compared to killing.^2^

We next used Bayesian model comparison to compare the predictor performance of variations of the model using the BayesFactor. To examine whether the variance in judgments of torture versus killing was best explained by mind perception, speciesism, or both, three Bayesian models were compared, which are variations on the model above, including all predictors or with only a subset of the predictors. The speciesism model contained only Species-Type (human vs. non-human) as a predictor; the Mind Perception model contained only Agency and Experience, and finally, the Full model contains all three predictors. The Mind Perception model substantially outperformed the speciesism model, *BF* = 1.89 × 1,019, suggesting that speciesism alone fails to account for the difference. However, the Full model substantially outperformed Mind Perception model, *BF* = 31.80, suggesting that speciesism has an important role over and above just mind perception in explaining judgments comparing extreme harms where torture seen as more clearly morally worse than killing for non-human animals, regardless of the degree of mind we ascribe to targets.^3^

### Discussion

Experiment 2 partially replicated and extended the results of Experiment 1. We found further support for the main prediction of an asymmetry in moral judgments of torture versus killing, for human versus animal targets. Torture was consistently rated as worse than killing for non-human animals, and killing was rated as worse for humans than for animals. But we also note that killing was not consistently rated worse than torture for human targets overall. One possibility is that by using specific examples of humans, rather than a generic “person,” participants were better able to simulate the experience and empathize with those particular targets. Nonetheless, there was a large difference between the nonhuman and human targets in relative ratings of torture versus killing, and judgments for humans were significantly more likely to say that killing is worse than judgments on animal targets.

The perceived minds of the targets partly explained these differences, and this interacted with Target-Type (human or animal). As predicted, higher perceived agency predicted judgments that of killing being worse compared to torture. In comparison, greater perceived experience predicted judgments that torture was seen as worse in comparison to killing. We further note that of the humans perceived to have less relative agency and greater experience (*baby, child, person with disability)*, the judgments of torture versus killing most closely resembled the moral preferences seen for the animal targets. These exemplars also appear to drive the mean ambivalence between torture and killing for human targets, when aggregating all human targets. This might be construed as a relative dehumanization of these people (Haslam & Loughnan, 2014), but we argue it more likely reflects a stronger empathy for those targets in imagining the experience of their torture (Gray & Wegner, 2010b). It is notable that judgments for the self (“you”), were also heavily skewed towards torture being worse, though judgments of own agency were among the highest among the human targets. Likewise, this may reflect a strong aversion in response to vividly imagining the experience of torture for oneself.

Moreover, we also found evidence that speciesism plays a role in judgments over and above mind perception. Torture was seen as morally worse than killing for non-human animals, regardless of how much more mind we ascribe to humans. In other words, there was greater value placed on human life overall, regardless of relative mind perception. It is clear that torture and killing are treated differently depending on whether the moral target is a human or an animal. In addition, we find evidence that mind perception mainly explains this, but that speciesism also plays a role.^4^

One limitation of this study design is that judgments regarding torture and judgments regarding killing cannot be distinguished since they are contrasted on the same scale. Therefore, it is not possible to measure the relative importance of dimensions of mind perception in making judgments of moral condemnation for each of these acts. Because of this in the following experiment, we separate judgments regarding killing from judgments regarding torture.

## Experiment 3

In our experiments so far, the relative moral wrongness of torture versus killing was consistently rated differently depending on whether the targets of extreme harms were humans or non-human animals. In both Experiments 1 and 2, participants were asked to judge whether torture or killing is worse for a given target, the key comparison being between torture and killing. But in pitting these extreme harms against each other it is unclear whether it is preferences for one kind or aversion to the other that drives judgments. In the final experiment, we changed to comparison to a decision between different targets while holding the kind of extreme harm (torture or killing) constant, to assess the role of mind perception and speciesism in moral judgments of each type of harm, separately. Because participants must now make multiple two-way comparisons between targets, we opted for only using a single human for comparison to prevent the experiment from being prohibitively long. Participants were assigned to one of two conditions (killing or harm judgments), and they rated the moral wrongness of these harms between pairs of targets (human, ant, dog, octopus, etc.) over several trials. They also rated the perceived capacities for agency and experience for each target. This study was pre-registered: https://osf.io/ce3kv/?view_only=451b6da65c9548038c43d114da7aeee9

### Participants

We recruited 163 participants (aged between 18 and 21, *M*_age_ = 18.66, 139 female, 20 male) from the psychology undergraduate student subject pool at the University of Warwick, in exchange for partial course credit. Post hoc sensitivity with α = .05 and 1 − β = .80, yielded a minimum detectable effect size of *d* = 0.07 (for a linear multiple regression with 4 predictors).

### Procedure

In a between-subjects design, participants were randomly assigned to make moral judgments about either killing (Killing condition) or torturing (Torture condition). Instructions for the Killing condition were as follows, with the variations for the Torture condition presented in brackets: *“We need your help! Two criminals are on the loose that have each captured pairs of 7 different animals and humans to kill [torture]. They intend to kill their victims painlessly [torture their victims painfully before letting them go]. Your task is to help us decide which is worse, killing [torturing] victim A or killing [torturing] victim B. You will be presented with 21 pairs of these and will answer questions on these pairings. For each one, imagine that this is the ONLY decision you have to make (i.e. no other killings [torturings] will take place).”*

Next, participants made judgments about pairs of targets, presented with all possible pairwise comparisons between the seven targets: orangutan, dog, cow, ant, pigeon, octopus, person. For each comparison, participants are asked four questions each on a five-point bipolar scales: (a) “Imagine you have the power to stop ONE of these criminals before they carry this out. Who do you stop?” with “Much prefer to stop criminal A” on one side of the scale, “Much prefer to stop criminal B” on the other and the central indifference point reading “Neither”; (b) “Which act of killing [torturing] is morally worse?”; with “Torturing Victim A is much worse” on one side of the scale, “Torturing Victim B is much worse” on the other, and the central indifference point reading “Both acts are the same”; (c) “Which criminal is more evil?” with “Criminal A is much more evil” on one side of the scale, “Criminal B is much more evil” on the other, and the central indifference point reading “Both are the same”; (d) “Imagine they both carry out the killing and are later caught by police. Both receive jail sentences, but one receives a much longer sentence. Who deserves the most punishment?” with “Criminal A deserves more punishment” on one side of the scale, “Criminal B deserves more punishment” on the other, and the central indifference point reading “They both deserve the same amount of punishment.”

Following all pairwise judgments, participants rated the perceived mind for each target, on a five-point scale, (endpoints 1 = “*not at all*”; 5 = “*extremely*”). Four items measured perceived agency: *“to what extent do you think [target]: “Is capable of thinking”; “Can plan its actions”; “Is intelligent,”; “Is able to think things through.”* Four items measured perceived experience: *“Is sensitive to pain”; “Can experience basic emotion (e.g., happiness, fear)”; “Can experience complex emotion (e.g., compassion, guilt)”; “Can experience empathy.”*

### Results

**Figure 4.**
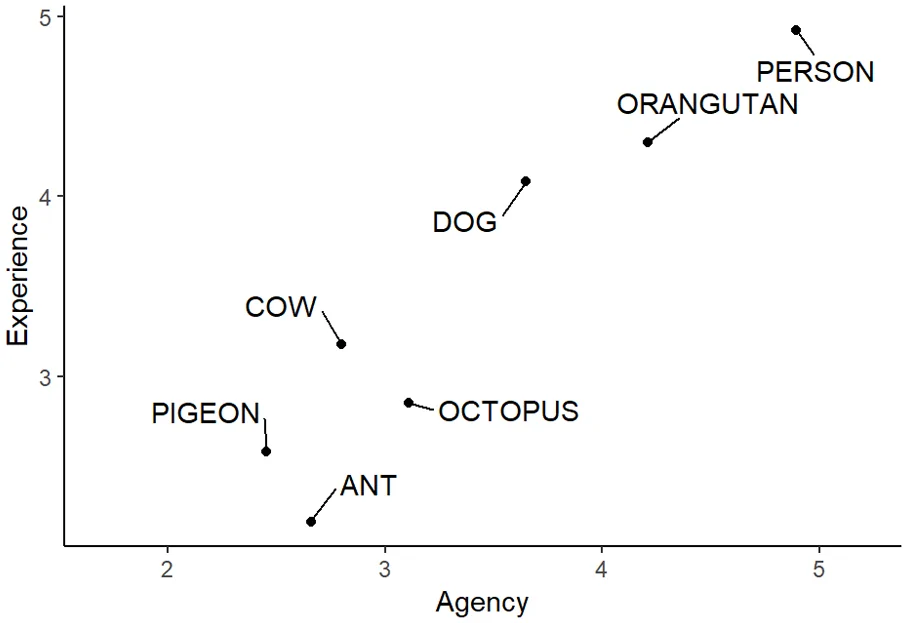
Mean perceived mind (agency/experience) for each target species, Experiment 3. *Note.* Note that again agency and experience ratings are highly correlated, *r*(161) = .82, *p* < .001.

To visualize how the targets differed in terms of mind perception, Agency and Experience are plotted for each target, see Figure 4.

A pre-registered reliability analysis across the four moral judgments (i.e., moral wrongness, stop, evil, and punishment) yielded a Cronbach’s α = .92 indicating that they can be averaged over for the following analyses. See Table 1 for means across mind perception and moral judgments for each group.

**Table 1.** Mind-Perception (Agency/Experience) and Moral Condemnation of Extreme Harms (Kill/Torture) by Species, Experiment 3.

Agent	Agency		Experience		Average Moral Condemnation			
					Torture		Killing	
	*M*	*SD*	*M*	*SD*	*M*	*SD*	*M*	*SD*
Person	4.89	0.28	4.92	0.22	+0.67	1.24	+0.85	0.92
Orangutan	4.21	0.60	4.30	0.64	+0.44	0.98	+0.53	.99
Dog	3.65	0.88	4.08	0.71	+0.35	0.99	+0.51	1.03
Octopus	3.11	1.06	2.85	0.91	−0.13	1.19	−0.21	1.02
Cow	2.80	0.90	3.17	0.83	−0.07	1.01	−0.22	0.99
Pigeon	2.45	0.97	2.58	0.89	−0.59	0.99	−0.50	1.00
Ant	2.66	1.09	2.19	0.97	−1.01	0.84	−1.01	0.85

Moral judgments were coded for each individual target by averaging comparisons made against every other target agent. Positive values indicate judgments were in favor of the target, and negative values indicate they were in favor of the comparison. Note that moral while moral judgments were aggregated, because mixed models were used there was no need to aggregate the data for comparisons between targets.^5^

A linear mixed model predicting average moral judgments was calculated with Target-Type (animal or human), and Group (Torture or Kill) and their interaction as predictors. Random intercepts were specified for participant ID and random slopes by participant were included for the effect of Target-Type.

**Figure 5.**
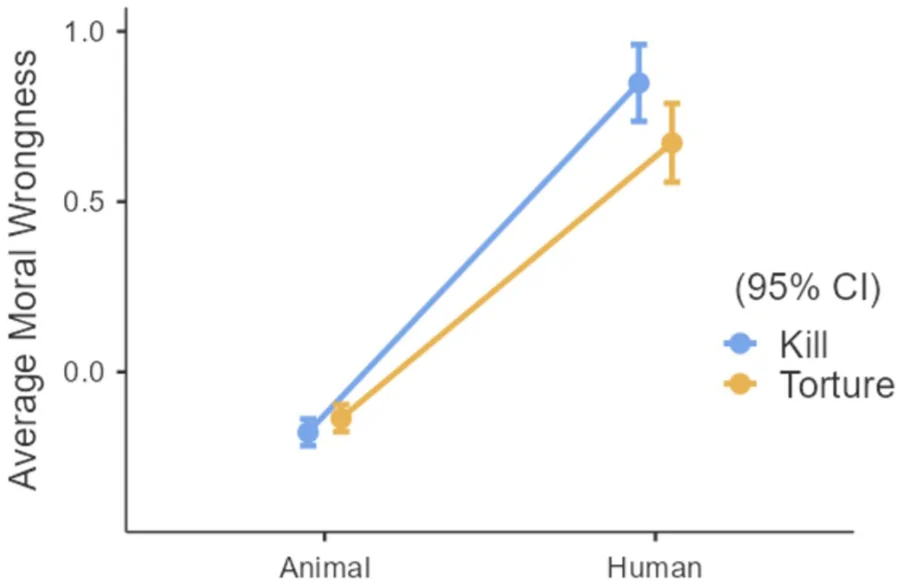
Moral wrongness of extreme harms by Target-Type (Animal vs. Human) and Harm-Type (Kill vs. Torture). *Note.* Bars indicate 95% CIs of the mean.

Results indicate a significant difference between Animal and Human targets, Target-Type: *F*(1, 161) = 403.62, *b* = 0.92, *SE* = 0.05, *p* < .001, *BF*_10_ = 1.34 × 10^102^, such that judgments were overall more severe with human targets. There was no significant difference between Torture and Kill groups, *F*(1, 299) = 2.74, *b* = −0.07, *SE* = 0.04, *p* = .099, *BF*_10_ = 0.053 with Bayesian evidence against this effect. Importantly, there was a significant interaction between Target-Type and Torture versus Kill Group, *F*(1,161) = 5.67, *b* = −0.22, *SE* = 0.09, *p* = .018, *BF*_10_ = 4.76 such that, as predicted, for judgments of killing it mattered more when the target was a human compared to nonhuman animals, compared judgments of torture, see Figure 5. This pattern indicates that when *participants compared animal targets, there was no apparent difference whether they were in the torture group or the killing group. However, when comparisons were made with human targets more moral condemnation was made in killing group than the torture group (although we note that this effect was quite small)*.

To assess the role of mind perception, a linear mixed model was calculated to predict average moral judgments with perceived Agency and Experience and their interactions as predictors, random intercepts for participants and random slopes for the predictors. Only Experience was significant, *F*(1, 81.2) = 403.86, *b* = 0.047, *SE* = 0.02, *p* < .001, *BF*_10_ = 1.73 × 10^108^. Neither Agency nor the interaction was significant: Agency *F*(1, 69.7) = 0.04, *b* = 0.01, *SE* = 0.02, *p* = .826, *BF*_10_ = 0.028, interaction; *F*(1, 1973.6) = 0.03, *b* = 0.01, *SE* = 0.01, *p* = .573, *BF*_10_ = 0.376.

In order to see how the Torture and Killing conditions differ from each other, we calculated a linear mixed model with group, mind-perception, and animal versus human as predictors (random intercepts for participant and random slopes for animal versus human and perceived experience—maximal converging model). Agency remained non-significant *F*(1, 2310) = 0.18, *b* = −0.01, *SE* = 0.02, *p* = .669. There was also no significant main effect of the torture versus killing groups, *F*(1, 147) = 1.05, *b* = −0.04, *SE* = 0.04, *p* = .307. Perceived experience significantly predicted judgments *F*(1, 339) = 338, *b* = 0.46, *SE* = 0.02, *p* < .001, such that higher experience predicted stronger moral condemnation. There was a significant difference between animal and human targets, *F*(1, 165) = 5.81, *b* = 0.12, *SE* = 0.05, *p* = .017. Lastly, there was a significant interaction between Target-Type and torture versus killing, *F*(1, 161) = 6.0, *b* = −0.21, *SE* = 0.08, *p* = .015, such that those the killing group gave stronger moral condemnation judgments compared to the opposite pattern for animals, however, note this effect was very small.

In addition, we also used Bayes factor model comparison analyzing the groups separately. For the torture condition, we compared two possible models, the first including just Experience and the second with both Experience and Target-Type. The model that included Target-Type failed to substantially improve on the experience-only model on its own *BF* = 0.19, (meaning there is substantial evidence that Target-Type should not be included). For the killing group, however, the model with Target-Type substantially improved on the experience-only model *BF* = 5.07 × 10^10^.

### Discussion

Experiment 3 examined judgments of killing or torture in separate conditions to examine the role of mind perception and speciesism in moral judgments of each extreme harm. More moral condemnation was given for human targets when they were in the killing group rather than the torture group, compared to animal targets, where the group did not seem to matter. We note, however, that this effect was quite small. The relative perceived experience of targets predicted the strength of moral judgments about both suffering and killing that target. Over and above the perceived ability to feel, it also mattered whether or not the target was human, especially for judgments about killing. In contrast, when making judgments about suffering it matters substantially less, whether the target is a human or animal if one accounts for the perceived experience of the target.

Evidence indicates that both experience and Target-Type together affect judgments about killing. In other words, for killing, whether or not the target is a human matter over and above mind perception (speciesism). However, for judgments of torture, the evidence is weaker for speciesism, consistent with the theory that there is speciesism for judgments about killing but not (or less so) for judgments about suffering. However, note that participants only made comparisons with a single human target. This limitation was required to prevent participants from having to make too many two-way comparisons.

We also find that, for human targets, killing is more immoral, evil and worthy of stopping than torture. But interestingly, we find that judgments around punishment for these acts show the opposite pattern. That is, people think that the torturer should be punished more harshly even though killing was judged worse. One potential explanation for this is that it is possible that the act of torture (compared to the act of killing) is perceived to be more diagnostic of the perpetrator’s character, such that we might predict they would continue to be harmful in future independent of any particular situational factors (Uhlmann et al., 2015). We note that the animal versus human comparisons in this study are more limited in scope compared to the previous study due to there being only a single human target for comparison.

## General Discussion

This research investigated lay judgments of a moral dichotomy: what is more morally wrong, to torture or to kill another being? People are generally averse to both the killing or suffering of others, and there may be no easy ordering of these extreme harms. Rather, the answer to this question appears to be strongly tied to the identity of the target. If the target is a human being, we may feel concern for their physical and emotional well-being, but we may also prioritize the preservation of life alongside these other concerns. In contrast, if the target is a non-human animal, we might prioritize their immediate physical welfare (i.e., that they aren’t suffering) far above how much value their life. In Study 1, people thought killing was morally worse for human targets, and in Study 2, although this asymmetry was not present, people were still more likely to say killing was worse for humans than they were for animal targets. It is important to note that one key difference between these two studies was that, while Study 1 specified the method of killing (i.e., “shooting painlessly in the head”), Study 2 only specified that it was a painless death without indicating exactly how the target was killed. This was done to avoid strange descriptions, such as shooting an ant through the head. Lastly, in Study 3, killing humans (compared to animals) was given more condemnation than torturing humans (compared to animals), although this effect was somewhat weaker. These preferences appear to be guided by a relationship between the role of speciesism (here anthropocentric speciesism, i.e., the feeling that humans are special) and mind perception (how we attribute different kinds of minds to different targets) when making moral judgments.

### Role of Mind Perception

First, the perception of the relative experience and agency of a target appears to play a critical role. As we assign complex mental and emotional states to beings, it becomes more challenging to justify any harm inflicted upon them. But as we assign greater agency to targets, the value of preserving life becomes a stronger concern, while perception of experience in targets elicits greater concern for avoiding suffering. Following the agency and experience model of mind perception (Gray et al., 2007, 2012; Gray & Wegner, 2009), we measured the extent to which participants ascribe mental capacities to the different targets. When people make comparisons between torture and killing, the higher the perceived agency (the ability for a target to think and act), the more killing is judged to be worse than torture. In contrast, higher perceived experience (the ability for a target to feel and experience positive and negative mental states) increased the judgment that torture was worse. This suggests that our ethical judgments towards animals are closely tied to our assumptions about their cognitive and emotional capacities. Ultimately, our moral compass seems to be guided not only by the object of our judgments but also by how we perceive their mental world. When considering killing and torture as extreme harms in isolation, perceived experience was consistently important for determining the severity of the harm. When it comes to suffering, it may be that we simply care about how much pain an agent is capable of feeling. It may be easier to imagine animals to be feeling beings rather than thinking beings, which may make animal suffering seem more pressing than animal death.

Although mind-perception predicted moral judgments, across the experiments, for each experiment, these measures did not fully explain variation in moral judgments that participants made between the targets, especially between animals and humans. This could suggest the mind-perception measures used here were weak proxies for the latent cognitive process, leaving unexplained variance. But we think the results more likely indicate that mind-perception only tells part of the story in judgments of extreme harm towards humans versus non-humans.

### Role of Speciesism

Second, anthropocentric speciesism may bias us towards both valuing human lives and human interests over those of non-human animals, and this bias can influence moral judgment over and above the degree of mind attributed to humans. In addition to mind perception, anthropocentric speciesism, the bias toward value of human life, (Caviola et al., 2019, 2020) made a difference over and above the kinds of mental abilities we attribute to humans compared to non-human animals where harm to humans is seen as worse. Importantly, this was specifically the case when making judgments about killing, where we are concerned about the sanctity of human life in general. However, when it comes to mitigating the suffering of different targets what matters most is simply how much we believe those targets are capable of suffering, and it appears there may be less of a role for species membership over and above this. This difference between moral judgments over animal lives compared to human lives has been highlighted in how far we appear to hold our deontological commitments. That is, it appears as if our lay ethics involve much stronger deontic constraints over human moral targets but we are more utilitarian about animals (Caviola et al., 2020; Nozick, 1974; Thompson, 1990). One of the reasons we may take such strong views on the loss of human life, is that human life is described as one of our “sacred values” (Tetlock, 2003) where it is deemed outrageous to trade human lives as if they were economic goods. For animal lives, it’s different. Animals are typically considered more in terms of economic value, and societal norms and cultural values may play a significant role in this. Likewise, our data supported a speciesism account in trade-offs between human and non-human targets.

Another mechanism not directly tested here is the differing social norms that exist between what is permissible for people versus non-human animals. It is non-normative to torture animals but there are many instances where the killing of animals is both normative and expected. Not only do people hunt animals for food but they also do so for sport, that is, “for fun.” By comparison, it is not normative to torture or kill humans, with very few exceptions. One aspect of the Theory of Dyadic Morality (Schein & Gray, 2018) is the use of comparison with a moral template of what a prototypical moral violation is. The fact that it may be easier to bring to mind such (normative) cases is likely to lead to less moral condemnation for acts that have some similarity to these cases. However, it may be hard to disentangle the independent roles of psychology (i.e., mind perception and speciesism) and the role of social norms. Indeed, the social norms regarding killing non-conspecifics may themselves originate from the very same psychological mechanisms, that it is more normative to subject animals to extreme harms because of biases toward our own species. However, one set of analyses that did pertain to this question was isolating farmed-from non-farmed animals. For both farmed and non-farmed animals, torture was seen as worse than killing but magnitude of this effect was larger for non-farmed animal, which would be expected on the account that social norms (with regard to farming) may explain the difference. However, including this predictor in the model did not explain away the effect of mind perception. Farming as a social norm is discussed in more detail below. Future work, both theoretical and empirical, could aim to distinguish these further. Further research may also include direct measures exploring the role of anthropocentric speciesism (e.g., Chandler & Dreger, 1993; Preston & Shin, 2021) to look for individual differences between people. If speciesism is operative for the kinds of moral condemnation judgments discussed here, then these individual differences will likely predict important differences in those judgments, and thus exploration of the role of speciesism can be more fine-grained.

### Implications

Our experiments asked participants to make moral judgments of an actor who committed extreme harms. However, the drivers of these judgments have wide potential implications for euthanasia, farming, and the rights and considerations of research subjects.

#### Euthanasia

In terms of practical implications, the present research informs how there is an important difference in how we view the humane ending of life for humans versus animals. Euthanasia is a highly controversial issue that has received considerable attention in both animal and human contexts. But while euthanasia for animals is widely accepted as a means of ending unnecessary suffering (Cholbi, 2017; Meijer, 2018), the practice of euthanasia for humans remains widely debated, raising complex moral and ethical issues, including judgments regarding the value of human life, the issue of consent, and the role of religion and culture in shaping attitudes toward death and dying Frileux, et al., 2003). Many people see animals as beings that do not have the same level of consciousness, self-awareness, and capacity for reasoning and decision-making as humans (Gray et al., 2007, 2012). As a result, they may view euthanasia for animals as a more merciful act, especially since animals themselves are not capable of making the decision to end their own lives. Indeed, the mercy killing of a suffering animal is often considered to be the most morally appropriate act, and suffering animals are put down routinely by veterinary practitioners (Cholbi, 2017; Meijer, 2018). In addition, humans are often seen as having a higher moral status than animals, and therefore, the decision to end a human life may be seen as a more significant moral issue (Caviola et al., 2019, 2020). This leaves us with a puzzle: humans are able to consent to euthanasia but animals are not, and yet the former is less likely to be judged as morally permissible than the latter. If human life is seen as inherently valuable then taking a life, even consensually in cases of extreme suffering, could be seen as morally wrong. Future applied work may also look at the kinds of arguments animal rights activists appeal to are more related to suffering than dying compared to arguments over concerns in human welfare.

#### Farming

As discussed above, separating out farmed versus non-farmed animals predicted people’s judgments. In the real world, judgments of the morality of suffering and death seem to change when it comes to animals, especially those considered as food sources (Leach et al., 2021). Views of animals as possessing less mind has been historically used to justify many acts that we would now consider barbaric, for example, vivisection without the use of anesthesia (Allen & Trestman, 2017). More recently, animal rights campaigners have drawn our attention to the suffering of animals under conditions of factory farming (Singer, 1995) leading to the widespread adoption of veganism (Gruen & Jones, 2015) or reductitarianism/flexitarianism (Derbyshire, 2016) (reduced meat consumption). In addition, prior to the 1990s in the United States, there was no official consensus on the existence of animal pain (in terms of phenomenological experience) and so veterinary practitioners were trained to ignore the signs of pain, thus withholding pain relief (Rollin, 1989). Animal lives under factory farming are often viewed as economic goods, and farms themselves emphasize humane ways of slaughter, focusing on the moral weight of causing undue suffering while treating animal death as a given. Causing death and causing suffering are qualitatively different kinds of harm, and the intention of this work was to see how judgments about these harms differ based on the different perceived characteristics of the moral target. Moreover, primary considerations for animals place greater value on their experience than the value of life, in raising animals for food, for example. Society generally condones practices that involve painless death for animals (e.g., euthanasia, “idealized” farming conditions). Greater perception of intelligent mind in nonhuman animals does predict the moral judgments against eating meat and toward vegetarian/vegan diets (Piazza & Loughnan, 2016), but people are often motivated to diminish animals’ minds in order to rationalize their own meat eating (Leach et al., 2022). Therefore, future work should aim at distinguishing the types of discourse prevalent in animal rights activism, which could lead to revealing insights on the ethical frameworks and emotional appeals that primarily drive these movements.

#### Rights and Considerations for Research Subjects

We may also further examine the ethical considerations for the treatment of human subjects or animal subjects in experimental research. For research conducted on animal subjects, there are strict guidelines to ensure the care and comfort of animals, suggesting an emphasis on their capacity for experience. For human subjects, the primary ethical concern is the question of informed consent, that is, does the subject know what they are getting into, and do they have the right to refuse or opt out? Here, the emphasis on informed choice is a clear value of human agency. Indeed, human subjects may even elect to engage in painful studies (e.g., shocks, ice water), if they do so by choice, indicating that their own agency overrides consideration of their experience. But non-humans are not given the same ethical consideration of choice to participate, only to minimize their discomfort in participation. Public opposition toward animal experimentation continues to grow (Goodman et al., 2012), and our understanding and recognition of animal consciousness has seen remarkable progress, evolving into mature fields encompassing animal cognition, neuroscience, and practical ethics (Allen & Trestman, 2017). But even these changes reflect a bias in that animal experience has gained more ethical concern than animal agency.

## Conclusion

In general, people are concerned about the suffering of animals and humans. But when it comes to moral judgments of torture versus death, animal and human lives are treated categorically differently, with death seeming a relatively worse harm to humans, and torture a relatively worse harm to animals. This difference seems to be explained in part by differences in perceived mind, with the relative capacity for experience in nonhumans prompting concerns for torture, and the relative capacity for agency in humans prompting concerns for killing. And beyond this, anthropocentric speciesism elevates the avoidance of the loss of human life, but much less so the avoidance of human suffering.

## Supplemental Material

sj-docx-1-psp-10.1177_01461672251367841 – Supplemental material for Avoiding Animal Suffering and Preserving Human Lives: Mind Perception and Speciesism in Moral Judgments of Torture and Killing Supplemental material, sj-docx-1-psp-10.1177_01461672251367841 for Avoiding Animal Suffering and Preserving Human Lives: Mind Perception and Speciesism in Moral Judgments of Torture and Killing by Simon Myers, Jesse L. Preston and Adam Sanborn in Personality and Social Psychology Bulletin

## Notes

1. Random effects indices and correlations are reported in the supplementary materials, as well as plots of random slopes of Target-Type for each participant, which indicate that not all participants showed the same response partner (direction and strength) for animals versus human targets.

2. As “*you”* is an unusual target, being directed at the self and not others, we performed a second calculation excluding “you.” Target-Type and Agency remain significant at the same level (*p* < .001), and Experience remains significant *p* = .002. The observed pattern for Agency and Experience remains the same. Thus, no qualitative differences are observed with and without “*You”* included.

3. In addition to these analyses, we also include frequentist model comparison of the same modes using the BICs. These are technically an estimation of a Bayes Factor (assuming unit information priors and assuming each parameter introduces the same amount of complexity to the model). These results concur with the main analyses.

4. We also replicate the procedure in Study 2, using a between-groups design, finding the same patterns. This study can be found in the Supplemental Material.

5. Note that when moral judgments are kept separate rather than aggregated, killing people is more immoral, evil, and worthy of stopping than torturing people. However, judgments around punishment for these acts show the opposite pattern (see Supplemental Material). That is, people think that the torturer should be punished more harshly. We return to this finding in the discussion.
