# Supplementary material for: Avoiding Animal Suffering and Preserving Human Lives: Mind Perception and Speciesism in Moral Judgments of Torture and Killing
Source: Pers Soc Psychol Bull. 2025 Oct 15;52(10):3310–23. doi: 10.1177/01461672251367841 (PMC13507505; doi:10.1177/01461672251367841)
Supplement: Supplementary file 1 [file sj-docx-1-psp-10.1177_01461672251367841.docx]

SUPPLEMENTARY MATERIAL

Study 2: Farmed vs Unfarmed Animals

Killing farmed animals painlessly should be expected to be somewhat normative, since this is what is expected to happen (not always painlessly) on farms. This alone could lead to the effect where ratings of torture being worse would be of a higher magnitude for animals. Therefore we included a predictor differentiating between the traditionally farmed animals (i.e. cows, sheep, chicken) and non-farmed animals, predicting ratings of torture vs killing (random intercepts for participant and target, and random slopes for farmed vs non-farmed). For both farmed and non-farmed animals, torture was seen as worse than killing but, as expected the magnitude of this effect was significantly stronger for non-farmed animal, F(1,14) = 14.50 , p = .002, b = -0.30 highlighting the potential role of social norms and expectations.

Next we included the measures of mind perception in the model (random intercepts for participant and target, and random slopes for farmed vs non-farmed, agency and experience) finding that these remained significant, agency F(1, 231.2) = 7.78, p = .006, b = 0.10; experience F(1,141) = 7.46, p = .007 , b = -0.12; but farmed was no longer significant F(1,19) = 3.70, p = .070 , b = -0.26. Taken together these results indicate that social norms do play a role in driving these judgments but isolating specific norms regarding farmed vs non-farmed animals did not explain away the effect. We return to this finding in theGeneral Discussion.

Study 2: Frequentist BIC Model Comparison

In the main text both frequentist models were tested and Bayesian model comparison was performed. Here, we also include frequentist model comparison using the BIC. These are technically an estimation of a Bayes Factor (assuming unit information priors and assuming each parameter introduces the same amount of complexity to the model). Three models were compared. The speciesism model contained only Species-Type (human vs. non-human) as a predictor, BIC = 11852; the Mind Perception model contained only Agency and Experience as predictors, BIC = 11253; and finally the Full model contained all three predictors, BIC = 10990. The results indicate that the full model performed the best and the Mind Perception model substantially outperformed the species-type model. These results suggest that speciesism alone fails to account for the difference in judgments. However, the Full Model substantially outperformed Mind Perception, suggesting that speciesism has an important role over and above just mind perception in explaining judgments comparing extreme harms where torture seen as more clearly morally worse than killing for non-human animals, regardless of the degree of mind we ascribe to targets.

Study 2: Random Effects Results

The random effects indices for Study 2 are presented in Tables S1 and & S2, and the random slopes have been plotted in Figure S1. The slopes reveal that participants do not all share the same pattern of responses when making judgments of animals compared to humans.

Table S1.

Random Effects Indices for Study 2

Table S2.

Random Parameter correlations for Study 2

Figure S1.

Random Slopes for Targets

Supplementary Experiment: Replication using a Between Groups Design

In a between-subjects design, participants made judgments for one of four different targets (2 humans/ 2 animals). We measure how participants perceive the minds of each target and test whether this predicts differences in judgment of torture vs killing. This study was pre-registered and can be found here: <https://osf.io/p7rcj/?view_only=4f9fa994a2e24f5aa50f376928a68c60>

Participants

We recruited 200 participants (aged between 18 and 62, Mage = 26.79, SDage = 8.59, 56% male) from Prolific Academic in exchange for a small payment of $2.40 USD.

Procedure

In a between-subjects design, participants were given examples of four targets. As there is a wide gap between humans and non-human animals in overall perceived mind (Gray, Gray, & Wegner, 2007), we wanted to provide examples where perceived mind varies in each human/ animal category. The four targets selected were: Cow, Orangutan, Professor, Person with Disability^[1]^.

Judgments of mind were measured with twelve questions on a five-point Likert scale. The agency questions were, “To what extent do you think the [target]: Can communicate with others, is capable of thinking, can plan its actions, is intelligent, has foresight, is able to think things through.” The experience questions were, “To what extent do you think the [target]: Is sensitive to pain, can experience happiness, can experience fear, can experience compassion, can experience empathy, can experience guilt”.

For measuring judgments of torture vs killing, participants are told: “Please imagine: First that someone, for fun, kills them instantly and painlessly. Second that someone, for fun, captures them and for a short period and painfully tortures them before letting them go.” Participants were asked which act is morally worse on a five-point scale between torture and killing (0 = torture is much worse, 5 = killing is much worse).

Results

First to assess whether the different targets differ in perceived mind, general linear models were fitted predicting agency and experience between each target. Analyses were performed using Jamovi v1.6 [(Şahin & Aybek, 2019)](https://paperpile.com/c/TUlpQE/LdE22). All targets significantly differed on judgments of Agency but only *Cow* differed on Experience, Cow: Agency; *b* = 1.75, *SE* = 0.14, *p* <.001, Experience; *b* = 0.13, *SE* = 0.14, *p* < .001; Orangutan, Agency: *b* = 0.82, *SE* = 0.14, *p* <.001; Experience: *b* = 0.16, *SE* = 0.13 , *p* = .239; Person with severe disability, Agency: *b* = 1.29, SE = 0.14, *p* < .001, Experience: *b* = 0.16 , *SE* = 0.13 , *p* = .239. Mind perception across agents is illustrated in the figure below.

Figure S2.

*Mind Perception (Agency/ Experience) of targets*

To test whether judgments of torture vs killing differed between targets, an independent-groups One-Way ANOVA revealed a significant difference in judgments of torture vs killing (*F* (3, 108.25) = 5.56, *p* = .001. Games-Howell post-hoc comparisons revealed that the following moral target pairs were significantly different from each other in relative judgments of extreme harms:, Cow - Professor (*p* = .03); Cow - Person with Disability (*p* = .006); Orangutan - Person with Disability (*p* = .017). There was no significant difference between the two animals nor between the two humans in relative judgments of extreme harms.

One-sample *t*-tests on the relative wrongness of torture vs. killing were calculated for each moral target. Both animal targets (*cow, orangutan)* significantly differed from the midpoint, falling on the “torture is worse side”, all *p*s < 0.001 (Bonferroni corrected). However, neither human target were significantly different from the midpoint (Person with Disability *p* = 0.118; Professor *p* = 0.070), meaning that participants did not rate either torture or killing as worse than the other for the human targets.

To test whether mind perception can account for these judgments, a General Linear Model predicting Torture vs Killing with Agency, Experience and Target-Type (Animal= 0; Human = 11) as predictors. Target-Type significantly predicted judgments, *F*(1,192) = 8.07, *B* = 0.82, *SE* = 0.29, *p* = .005, BF_10_ =262.96;. However, all other predictors were non-significant, Agency, *F*(1,192) = 0.002, *B* =-0.01, *SE* = 0.18, *p* =0.964, BF_10_ = 0.20; Experience, *F*(1,192) = 00.02, *B* =-0.03, *SE* = 0.24, *p* = .887, BF_10_ = 0.17, see Figure 3

The same model only testing mind perception (not including target type) also failed to find that Agency was a significant predictor *F*(1,197) = 2.04, *B* =0.13, *SE* = 0.17, *p* = .115; nor Experience *F*(1,197) = 0.05, *B* =-0.02, *SE* = 0.22, *p* = .831.

Although judgments, on average fell on the “torture is worse” side, to test whether people were more likely to choose killing is worse for humans than animals we re-coded the dependent variable dichotomously as either “killing is worse” (3 - 5) or “torture is worse (0 - 3). A generalized linear regression was performed to predict recoded torture vs killing judgments with Target-Type (animal or human) as a predictor. Results confirm that participants were significantly more likely to choose “killing is worse” for humans than for animals χ^2^(1) = 11.88, β = 1.16, *SE* = 0.35, *p* < 0.001. Twice as many people thought killing was worse for humans than for animals.

Figure S3.

*Moral preferences of Torture vs Murder by target*

*Note: Top: Dots are jittered data points behind violin (distribution) and box plots.*

**Experiment 3 Supplemental Material**

Rather than being averaged, when moral judgments are separated the punishment judgments show an interesting pattern (see footnote 3 in manuscript). Results show that killing people is more immoral, evil and worthy of stopping than torturing people. However, judgments around punishment for these acts show the opposite pattern (see Figure S3). In other words people think that the torturer should be punished more harshly. This finding is elaborated on in the discussion. Results were as follows, Stop: Results indicate a significant difference between Animal and Human targets, *F*(1,7983) = 615.83, *b* =-0.14, *SE* = 0.05, *p* < .001 and a significant difference between Torture and Kill groups *F*(1, 7983) = 8.79, *b* =1.13 *SE* = 0.05, *p* = .003. There was a significant interaction between Target-Type and Torture vs Kill Group, *F*(1,7983) = 22.43, *b* = -0.43, *SE* = 0.09, *p* < .001. Moral: Results indicate a significant difference between Animal and Human targets, *F*(1,7983) = 642.6, *b* =0.97, *SE* = 0.04, *p* < .001 and a significant difference between Torture and Kill groups *F*(1, 7983) = 17.3, *b* =-0.16, *SE* =0.04, *p* < .001. There was a significant interaction between Target-Type and Torture vs Kill Group, *F*(1,7983) = 40.4, *b* = -0.48, *SE* = 0.08, *p* < .001. Evil: Results indicate a significant difference between Animal and Human targets, *F*(1,7983) = 570.1, *b* =0.88, *SE* = 0.04, *p* < .001 and a significant difference between Torture and Kill groups *F*(1, 7983) = 11.0, *b* =-0.12, *SE* =0.04, *p* < .001. There was a significant interaction between Target-Type and Torture vs Kill Group, *F*(1,7983) = 26.4, *b* = -0.38, *SE* = 0.07 *p* < .001. Punish: Results indicate a significant difference between Animal and Human targets, *F*(1,7983) = 300.1, *b* =0.69, *SE* = 0.04, *p* < .001 and a significant difference between Torture and Kill groups *F*(1, 7983) =13.6, *b* =0.14, *SE* =0.04, *p* < .001. There was a significant interaction between Target-Type and Torture vs Kill Group, *F*(1,7983) = 28.4, *b* = 0.43, *SE* = 0.08, *p* < .001.

Figure S4.

*Separated Moral Wrongness judgments*

1. While Professor and Person with Severe Mental Disability are overlapping categories, this target was chosen to allow for the possibility that there would be an adult human for whom people may differ in the kind of mind they would perceive.course.
